# Status and correlates of children’s exposure to secondhand smoke at home: A survey in Chongqing, China

**DOI:** 10.18332/tid/159802

**Published:** 2023-03-13

**Authors:** Longxian Huang, Yang Cao, Zhiyong Zhang, Ya Zhang, Mei Kuang, Yetao Luo, Li Zhang

**Affiliations:** 1 Respiratory Medicine Department, The First Branch of the First Affiliated Hospital of Chongqing Medical University, Chongqing, People's Republic of China; 2 Health Center of Bafu Town, Bafu Town, People's Republic of China; 3 Integrated Traditional Chinese Medicine and Western Medicine Department, Healthcare Center, Jinlong Town, Chongqing, People's Republic of China; 4 Nursing Department, Shiqiaopu Street Healthcare Center, Chongqing, People's Republic of China; 5 Nursing Department, Township Hospital, Jinfeng Town, People's Republic of China; 6 Clinical Epidemiology and Biostatistics Department, Children’s Hospital, Chongqing Medical University, Chongqing, People's Republic of China; 7 College of Nursing, Chongqing Medical University, Chongqing, People’s Republic of China

**Keywords:** children’s secondhand smoke exposure, environmental tobacco smoke, passive smoking, tobacco control services, smoke-free home

## Abstract

**INTRODUCTION:**

The home is the primary source of children’s exposure to secondhand smoke. This study investigated the status and influencing factors of child exposure to secondhand smoke at home when people smoke in the household.

**METHODS:**

Participants with at least one child living in their household from 10 communities in Chongqing were recruited and provided a self-administered questionnaire using a multistage proportional random sampling design from June to August 2021. The chi-squared test and binary logistic regression analyses were used to identify influencing factors.

**RESULTS:**

The questionnaire completed by 1345 families showed that 631 (46.9%) families lived with smokers in their household, and 509 (80.7%) of those families reported that smoking occurred within the home while the children were present. Binary logistic regression analyses demonstrated that the time between waking up and household smokers having the first cigarette of the day (OR=0.44; 95% CI: 0.22–0.85), changes to smoking habits and behaviors within the last six months (OR=1.76; 95% CI: 1.06–2.90), attitudes towards tobacco control in the household (OR=2.91; 95% CI: 1.72–4.92), self-efficacy in maintaining a smoke-free home (OR=2.27; 95% CI: 1.36–3.79), having rules to maintain a smoke-free home (OR=3.25; 95% CI: 1.68–6.29), and the status of providing cigarettes to guests at home (OR=11.0; 95% CI: 1.33–90.8) were associated with exposure to SHS.

**CONCLUSIONS:**

Education focusing on the impact of smoking on children’s health should be encouraged. Smoke-free homes should be established, and smoking restrictions in the household should be enacted. Therefore, information about the available tobacco-control services should be given to family members and be used properly. It is an effective way to decrease the risk of at-home exposure to SHS for children, to overcome any obstacles in tobacco control.

## INTRODUCTION

Secondhand smoke (SHS) has been associated with coronary heart diseases, cancers, respiratory diseases, and other health problems^1^. No safe level of exposure to secondhand smoke generated by tobacco products exists or has been reported. Even inhaling passive smoke generated by tobacco products for a short time can have a detrimental effect on a person’s health^2^. According to WHO, over one million people have died from SHS^2^. Children are particularly vulnerable to SHS^3,4^. Exposure to the toxic substances in SHS during childhood may inhibit nerve growth and lead to behavioral problems^5^. Studies have shown that SHS affects lung function of children and leads to respiratory symptoms such as coughing, asthma, and bronchitis^6^. In addition, SHS increases the risk of cancer^7^ and cardiovascular disease^8^ within a person’s lifetime. Exposure to SHS may also contribute to the likelihood of a child becoming a smoker in the future^9^.

China is the largest producer and consumer of tobacco, with 316 million smokers, and according to Global Adult Tobacco Survey Fact Sheet, 68.1% of non-smokers have been exposed to SHS^10^. Recent studies have reported that 66.7% of children aged <15 years live in a household with active smokers, and among them, 180 million children have reportedly suffered from exposure to SHS^11^. The prevalence of exposure to SHS was the highest for children, in 21 countries/regions^12^. To reduce the negative impact of SHS, smoke-free regulations have been enforced in public areas nationwide. Thus, rates of exposure to SHS in public areas have been declining^10^. However, households have been considered private spaces, therefore, they have not been regulated by the same tobacco-control legislation that applies to public areas. Previous studies have revealed that the home became the primary place for people to smoke after smoke-free legislation banning smoking in public spaces. As a result, family members, especially children in the same household as smokers, have become more vulnerable to exposure to SHS^13^.

Parents and family members who smoke and how they smoke at home play a key role in reducing children’s exposure to SHS^13,14^. Different regions of China have varied risk factors and reasons associated with their rates of at-home SHS exposure for children^15^. Research has affirmed that the highest rates of at-home SHS exposure for children can be found in southwest China^16^. This study aimed to identify the influencing factors and the prevalence of at-home SHS exposure of children living in Chongqing, China, so that regional evidence can be provided to indicate the necessary measures to reduce it.

## METHODS

### Study design

The study was one of the components of a family tobacco control study after the implementation of a regional smoke-free legislation in public areas. As the methods had been previously described in detail^17^, we elaborated them here briefly. Participants from 10 community healthcare service centers of different regions in Chongqing were recruited using a random sampling design from June to August 2021. The self-administered questionnaire regarding home tobacco control was collected while they were vaccinated against COVID-19 during the 30-minute waiting time. After the introduction of the purpose of the survey, participants aged >18 years who lived with a family with a child aged <14 years completed the questionnaire. Written informed consent was obtained from each participant. To avoid duplicate responses, the questionnaire was sent to only one person for each family, and an IP address could only submit once.

### Questionnaire

A self-administered questionnaire based on the knowledge, attitudes and practices model was used after reviewing the literature^13,18-21^ and discussing best practices with experts. Cronbach’s alpha of the questionnaire was 0.806, affirming the internal consistency of the tool. The questionnaire was composed of the following four sections:

Demographic factors of the participant: gender, age, education level, occupation, confirmation that they lived in the same household with children aged <14 years, and the smoking status of family members at home.

Knowledge of tobacco control: 1) A total of 11 questions about the hazards of secondhand smoke with score ranging 0–11. If a response was correct, one point was given; 2) A total of 5 questions about tobacco control and cessation services, including clinics, hot lines, apps (i.e. APP), medications, and websites. Responses were provided on a Likert scale of 1 = ‘don’t know at all’ to 5 = ‘ever used’, with a score ranging 5–25.

Attitudes towards tobacco control in the household (5 questions) included the importance of no smoking at home and self-efficacy, awareness of the tobacco hazard to children’s health, and four aspects of smoking restrictions in the household (smoking regulations at home and in the cars, the status of providing cigarettes to the guests at home, and using cigarettes as gifts to others).

Outcome variable: SHS exposure was defined as non-smokers ever exposed to the passive smoke from burning cigarettes or smoke exhaled by smokers at least one day in a week^22^. As previous research has shown that the concentration of tobacco metabolites in the urine of children with an active smoker in the household is higher than those in a non-smoking home^14^, we asked: ‘What is the frequency of you/your family member smoking at home in a week when a child is present?’. The response options were: 0, 1–3, 4–6, and 7 days. In the study, 0 days was defined as non-exposure to SHS of children at home, while all other options were defined as exposure to SHS of children at home.

### Statistical analysis

Descriptive analyses were performed and reported as mean ± standard deviation or frequency and percentage. The chi-squared test was used for the assessment of differences of at-home SHS exposure of children. All significant variables in the bivariate level were entered into a binary logistic regression model. A p<0.05 was considered statistically significant. SPSS 20.0 (IBM Corporation, Armonk, NY, U.S.) was used for the data analysis

## RESULTS

### Status of children’s exposure to SHS at home

Of the 1345 participants living with children aged <14 years included in the 2121 families who took part in the primary study, 631 families (46.9%) reported that there were smokers that lived in the household and in those families, 509 (80.7%) reported that they or someone else they lived with smoked in the household at least once per week when a child was present, accounting for 38.8% of the total families with children (509/1345) (Table 1).

**Table 1:** Children’s exposure to SHS at home, Chongqing, China, 2021 (N=631)

*Days in a week when you/your family members smoke in the presence of children at home*	*n*	*%*
0	122	19.3
1–3	187	29.6
4–6	47	9.0
7	265	42.0

### Participant characteristics related to at-home SHS exposure of children

Among the 631 participants who were or lived with smokers, 249 (39.5%) were male, and 382 (60.5%) were female. Age ranged from 21 to 75 years, with an average age of 37.9 ± 9.7 years. Regarding education level, 393 (62.3%) participants reported that they did not complete high school. Moreover, 383 participants (60.7%) were unemployed or farmers. There were no significant differences between participant demographic characteristics (gender, age, education level, occupation, the number of children) and at-home SHS exposure of children when the children were at home (p>0.05). However, results did indicate a significant difference among the risk of household SHS exposure of children with cigarette consumption, the amount of time between waking up and a household smoker having the first cigarette of the day, and if smoking behaviors had changed in the last six months (p<0.05) (Table 2).

**Table 2:** Characteristics of participants and children’s exposure to SHS at home, Chongqing, China, 2021 (N=631)

*Characteristics*	*Total n (%)*	*Exposure n (%)*	*Non-exposure n (%)*	*χ* ^2^	*p*
**Total**	631 (100)	509 (80.7)	122 (19.3)		
**Gender**				0.147	0.702
Male	249 (39.5)	199 (79.9)	50 (20.1)		
Female	382 (60.5)	310 (81.2)	72 (18.8)		
**Age** (years)				3.541	0.315
≤30	83 (13.2)	66 (79.5)	17 (20.5)		
31–40	348 (55.2)	275 (79.0)	73 (21.0)		
41–50	130 (20.6)	106 (81.5)	24 (18.5)		
≥51	70 (11.1)	62 (88.6)	8 (11.4)		
**Education level**				3.904	0.419
Primary school	68 (10.8)	58 (85.3)	10 (14.7)		
Middle school	146 (23.1)	122 (83.6)	24 (16.4)		
High school	179 (28.4)	144 (80.4)	35 (19.6)		
College	116 (18.4)	93 (80.2)	23 (19.8)		
Undergraduate or higher	122 (19.3)	92 (75.4)	30 (24.6)		
**Occupation**				1.160	0.763
Leader of enterprise or institution	38 (6.0)	30 (78.9)	8 (21.1)		
Professional technician	106 (16.8)	82 (77.4)	24 (22.6)		
Service employee	104 (16.5)	86 (82.7)	18 (17.3)		
Farmer or unemployed	383 (60.7)	311 (81.2)	72 (18.8)		
**Number of children in the family aged <14 years**				0.867	0.648
1	386 (61.2)	307 (79.5)	79 (20.5)		
2	208 (33.0)	171 (82.2)	37 (17.8)		
≥3	37 (5.9)	31 (83.8)	6 (16.2)		
**Your/your family’s daily cigarette consumption**				28.281	0.000
≤10	314 (49.8)	227 (72.3)	87 (27.7)		
11–20	238 (37.7)	213 (89.5)	25 (10.5)		
21–30	62 (9.8)	54 (87.1)	8 (12.9)		
≥31	17 (2.7)	15 (88.2)	2 (11.8)		
**How long after waking in the morning do you/your family members smoke the first cigarette?** (minutes)				37.520	0.000
≤5	158 (25.0)	141 (89.2)	17 (10.8)		
6–30	201 (31.9)	173 (86.1)	28 (13.9)		
31–60	91 (14.4)	76 (83.5)	15 (16.5)		
>60	181 (28.7)	119 (65.7)	62 (34.3)		
**Status of your/your family members’ smoking behavior in the last six months?**				7.452	0.024
Increased	59 (9.4)	55 (93.2)	4 (6.8)		
No difference	360 (57.1)	290 (80.6)	70 (19.4)		
Decreased	212 (33.6)	164 (77.4)	48 (22.6)		

### Parental knowledge, attitudes to tobacco control related to at-home SHS exposure of children

The highest score relating to the harmful effects of at-home SHS exposure of children was 11, with an average of 6.65 ± 3.81. The highest score relating to the participant’s knowledge of tobacco-control services was 23/25. High scores and low scores were divided according to an average of 8.9 ± 3.2. The statistics showed no meaningful difference due to parental knowledge of tobacco control services (p>0.05). However, 89.1% of families were concerned that SHS was hazardous to the children’s health; 89.7% of participants supported that it is important to have a smoking ban at home and 45.6% agreed it was difficult to stop smoking inside the home. Only 35% of participants felt confident that they could stop others from smoking in the home. This demonstrated that parental attitudes towards tobacco control in the household were associated with the rate of at-home SHS exposure of children, and the difference was statistically significant (p<0.05) (Table 3).

**Table 3:** Analysis of parental knowledge, attitudes to tobacco control in relation to children’s exposure to SHS at home, Chongqing, China, 2021 (N=631)

*Variables*	*Total n (%)*	*Exposure n (%)*	*Non-exposure n (%)*	*χ* ^2^	*p*
**Total**	631 (100)	509 (80.7)	122 (19.3)		
**Score of harmful effects of SHS**				0.074	0.785
Low score (1–7)	338 (53.6)	274 (81.1)	64 (18.9)		
High score (8–11)	293 (46.4)	235 (80.2)	58 (19.8)		
**Score of knowing about cessation services**				0.004	0.949
Low score (5–9)	288 (45.6)	232 (80.6)	56 (19.4)		
High score (11–23)	343 (54.4)	277 (80.8)	66 (19.2)		
**Are you concerned about the health of children when you or others smoke in the household?**				1.966	0.191
Concerned	562 (89.1)	449 (79.9)	113 (20.1)	3.638	0.162
Not concerned at all	69 (10.9)	60 (87.0)	9 (13.0)		
**Are you concerned about whether your children become smokers in the future?**				0.065	0.798
Concerned	512 (81.1)	414 (80.9)	98 (19.1)		
Not concerned at all	119 (18.9)	95 (79.8)	24 (20.2)		
**Do you find it important to have smoking restrictions at home?**				6.298	0.012
Important	566 (89.7)	449 (79.3)	117 (20.7)		
Not important at all	65 (10.3)	45 (92.5)	5 (7.7)		
**Do you find it difficult to maintain a smoke-free household?**				47.122	0.000
Difficult	326 (51.7)	297 (91.1)	29 (8.9)		
No difficult	305 (48.3)	212 (69.5)	93 (30.5)		
**How confident are you about preventing others from smoking in the household?**				58.743	0.000
Confident	221 (35.0)	142 (64.3)	79 (35.7)		
Not confident	410 (65.0)	367 (89.5)	43 (10.5)		

### Smoke-free home policy in relation to the rate of at-home SHS exposure of children

The study showed that there were 201 families (31.9%) who reported that they maintained a smoke-free home, 426 families (67.5%) reported that they maintained a smoke-free car, 89 families (14.1%) reported that they never offered cigarettes to guests, and 252 families (39.9%) never provide cigarettes as a gift to guests. Maintaining a smoke-free home was statistically significant with regard to the rate of at-home SHS exposure of children (p<0.05) (Table 4).

**Table 4:** Analysis of maintaining a smoke-free home and children’s exposure to SHS at home, Chongqing, China, 2021 (N=631)

*Variables*	*Total n (%)*	*Exposure n (%)*	*Non-exposure n (%)*	*χ* ^2^	*p*
**Total**	631 (100)	509 (80.7)	122 (19.3)		
**What restrictions or rules do you have for smoking at home (e.g. smoke in an open balcony)?**				36.169	0.000
Smoking allowed anywhere inside the house	60 (9.5)	54 (90.0)	6 (10.0)		
Smoking allowed in some rooms inside the house	191 (30.3)	176 (92.1)	15 (7.9)		
Smoking not allowed indoor except for some special circumstances	179 (28.4)	139 (77.7)	40 (22.3)		
Smoking not allowed in the household	201 (31.9)	140 (69.7)	61 (30.3)		
**What restrictions or rules do you have for smoking in your car when a child is present?**				14.961	0.002
Smoking never allowed in any car	426 (67.5)	328 (77.0)	98 (23.0)		
Smoking allowed sometimes or in some cars	129 (20.4)	109 (84.5)	20 (15.5)		
Smoking allowed in the car	18 (2.9)	18 (100)	0 (0.0)		
No cars available in the family	58 (9.2)	54 (93.1)	4 (6.9)		
**What is the status of providing cigarettes to guests?**				44.572	0.000
Never	89 (14.1)	51 (57.3)	38 (42.7)		
Occasionally	242 (38.4)	194 (80.2)	48 (19.8)		
Sometimes	161 (25.5)	138 (85.7)	23 (14.3)		
Often	97 (15.4)	5 (87.6)	12 (12.4)		
Always	42 (6.7)	41 (97.6)	1 (2.4)		
**What is the status of using cigarettes as gifts to others?**				2.747	0.601
Never	252 (39.9)	201 (79.8)	51 (20.2)		
Occasionally	247 (39.1)	195 (78.9)	52 (21.1)		
Sometimes	102 (16.2)	87 (85.3)	15 (14.7)		
Often	24 (3.8)	21 (87.5)	3 (12.5)		
Always	6 (1.0)	5 (83.3)	1 (16.7)		

### Logistics regression analysis of at-home SHS exposure of children

SHS exposure of children at home was defined as family members smoking in the household in the presence of children per week. With the rate of at-home SHS exposure of children as the dependent variable (exposure=1, non-exposure=0) and the nine factors that were significant in the bivariate analysis (cigarette consumption, the amount of time between waking up and a household smoker having the first cigarette of the day, changes in smoking behaviors during the last six months, the attitudes and self-efficacy about tobacco control in the household, and maintaining a smoke-free home and car), a binary logistic regression analysis was performed. According to Table 5, the longer the time between waking up and a household smoker having the first cigarette of the day, the lower the risk of household SHS exposure of children (OR=0.44; 95% CI: 0.22–0.85), while increasing cigarette consumption in the previous six months increased the risk of household SHS exposure of children by 1.76 times (OR=1.76; 95% CI: 1.06–2.9). SHS exposure was 2.9 times higher for children in families where it was difficult to maintain a smoke-free household (OR=2.90; 95 % CI: 1.72–4.92); SHS exposure was 2.27 times higher for children in families where there was no confidence to maintain a smoke-free household (OR=2.27; 95% CI: 1.36–3.78). The risk of at-home SHS exposure of children in households that never provided cigarettes to guests was 2.68 times lower than those who always provided cigarettes to guests (OR=2.68; 95% CI: 1.13–6.36) (Table 5).

**Table 5:** Binary logistic regression of the factors influencing children’s exposure to SHS at home, Chongqing, China, 2021 (N=631)

*Variables*	*B*	*S.E.*	*Wals χ^2^*	*p*	*OR*	*95 % CI*
**Do you find it difficult to maintain a smoke-free household?**						
Difficult (Ref.)						
Not difficult	1.067	0.269	15.788	0.000	2.91	1.72–4.92
**How confident are you about preventing others from smoking in the household?**						
Confident (Ref.)						
Not confident	0.819	0.262	9.792	0.002	2.27	1.36–3.79
**What restrictions or rules do you have for smoking at home (e.g. smoking in an open balcony)?**						
Smoking not allowed anytime in the household (Ref.)						
Smoking is not allowed indoors except for some special circumstances	-0.095	0.274	0.121	0.728	0.91	0.53–1.56
Smoking is allowed in some rooms inside the house	0.124	0.542	0.053	0.819	1.13	0.39–3.28
Smoking is allowed anywhere inside the house	1.179	0.337	12.234	0.000	3.25	1.68–6.29
**What’s the status of providing cigarettes to guests in the household?**						
Never (Ref.)						
Occasionally	0.846	0.312	7.371	0.007	2.33	1.27–4.30
Sometimes	1.142	0.359	10.136	0.001	3.14	1.55–6.33
Often	0.986	0.441	4.995	0.025	2.68	1.13–6.36
Always	2.398	1.077	4.957	0.026	11.00	1.33–90.78
**Time after waking in the morning before smokers have their first cigarette** (minutes)						
≤5 (Ref.)						
6–30	-0.016	0.355	0.002	0.964	0.98	0.49–1.98
31–60	0.063	0.428	0.022	0.882	1.07	0.46–2.46
>60	-0.830	0.342	5.907	0.015	0.44	0.22–0.85
**Changes in smoking habits and behaviors within the last six months**						
Increased (Ref.)						
No changes	1.112	0.589	3.566	0.059	3.04	0.96–9.65
Decreased	0.564	0.256	4.848	0.028	1.76	1.06–2.90

## DISCUSSION

Other peoples’ smoking in the home, including that of parents, is a precursor to more toxic chemical inhalation by children^23^. This study estimated that 37.8% of children were exposed to SHS at home in Chongqing, which was higher than the reported level in the US (21.7%)^24^. One of the possible explanations for this difference may be the adoption of smoke-free policies and diverse tobacco-control education. It is lower than the reported level of SHS for children in Macao after legislation of a smoke-free policy (41.3%)^13^, and even lower than the level in other areas in China (41.7–47.3%)^16^, which may be related to the awareness of tobacco control, which may have strengthened for the public after the implementation of smoke-free policies in some areas. On the other hand, we limited our study to participants living with children aged <14 years, whereas previous research included children aged 14–18 years and hence may reflect different populations, as research has indicated that the rate of at-home SHS exposure of children aged 14–18 years was higher than for those who were younger^16,25^. Theoretically, the number of children in the family may be associated with the rate of children’s exposure to SHS at home. However, we found no statistical difference, which may be related to the two-children or three-children birth policy in China that has been implemented in recent years. The majority of our participants were one-child families (61.2%).

In this study, the highest rate of at-home SHS exposure of children was correlated with a smoker having their first cigarette of the day in 60 minutes after waking up and how much they smoked in the household. This may reflect the tobacco dependence of smokers. This short window between awakening and lighting their first cigarette indicates a stronger addiction to tobacco products^26^. Furthermore, we found that the increased cigarette consumption in the last six months was associated with exposure to SHS for children in the household. Fortunately, smoke-free legislation in public spaces was implemented half a year before the investigation, and hence only 9.35% of participants reported that they had increased cigarette consumption, whereas 33.6% of participants reported that they had decreased their consumption. Meanwhile, it demonstrated that higher rates of at-home SHS exposure of children were associated with the difficulty in asking others not to smoke in their home and a lack of confidence in smoke-free home restrictions. This finding is consistent with the Health Belief model. Smokers who decreased dependence on tobacco products were directly correlated with a lower rate of at-home SHS exposure of children. Therefore, it may play an important role in overcoming the difficulties of establishing a smoke-free home and improving the self-efficacy of family members in enforcing smoking restrictions for smokers in the household. The statistics revealed that smokers and family members knew little about smoking cessation services. At least in part, it showed that most Chinese smokers who quit smoking did so as a result of perseverance on their own, not as a result of using smoking cessation aids or services^27^. Currently, smoking cessation websites, clinics, and hotlines as well as medications for smoking cessation, are available for smokers^28^. This has not, however, led to an improvement in smoking cessation education, which has in turn, resulted in a lack of knowledge regarding these services^29^. All of which suggests that more efforts should be made to increase educational opportunities about the harms of smoking and promote the benefits of smoking cessation for individuals and their communities. To improve the rates of tobacco-control confidence at home, smokers and their family members need to have a special focus of these educational efforts, and improved self-efficacy in maintaining a smoke-free home and smoking cessation overall, should be the targets to determine the success of such efforts.

The data confirmed that the risk of at-home SHS exposure of children increased significantly when there was no smoke-free policy in the household and guests in the home were offered cigarettes. It is considered a sign of hospitality in Chinese homes to politely offer cigarettes to guests^30^; thus, it is a common phenomenon. As a result, they typically smoke in the presence of children. To address this issue, we must raise awareness of the dangers of tobacco products, as well as the dangers and health risks of secondhand and firsthand smoke, and we must consider ways to promote healthier habits^31^. Moreover, initiatives focused on creating and maintaining a smoke-free home, regardless of the presence of smokers as guests or residents in the home, are necessary to protect the health of children.

### Limitations

This study for the first time investigated the status and influence factors of children’s exposure to SHS at home after smoke-free legislation in public areas and workplaces in Chongqing, providing the reference for tobacco control in homes. It has, however, certain limitations. The investigation relied on self-reported data on smoking behavior in the presence of children to determine the risk of at-home SHS exposure of children. Social expectations and customs regarding smoking habits and behaviors may have influenced the honesty and comfort of participants when filling in the questionnaire, leading to report bias. Second, the study was based on self-reported data and, therefore, lacked objective biochemical parameters. It has been well-documented that the levels of nicotine metabolites in the urine of children living in households with smokers are significantly higher than those in non-smoking homes^23^. In future research, biochemical parameters and indices should be collected. Third, the sample comprised data related to smoking in the presence of children but did not account for smoking habits when the children were not around. Fourth, we can only associate factors and make suppositions, owing to the nature of the cross-sectional study. Finally, as the study was conducted in Chongqing, the rate of at-home SHS exposure for children cannot be generalized to the population of China.

## CONCLUSIONS

Smokers’ high dependence on tobacco products was linked to smoking in the home. To effectively prevent children from being exposed to SHS at home, efforts to educate the public about the harmful effects of smoking on children’s health, as well as their own, should be increased. Smoke-free homes and smoking-free environments should be established. Family members of smokers should be given information and guidance on smoking cessation services and tools. Furthermore, self-efficacy in establishing and maintaining a smoke-free home should be encouraged.

## Data Availability

The data supporting this research are available from the authors on reasonable request.

## References

[1] World Health Organization Tobacco.

[2] Al-Sayed EM, Ibrahim KS (2014). Second-hand tobacco smoke and children. Toxicol Ind Health.

[3] Kang SH, Joo JH, Jang SI, Park EC (2020). Association of exposure to secondhand smoke at home with early age at menarche in South Korea. Public Health.

[4] Homa DM, Neff LJ, King BA (2015). Vital signs: disparities in nonsmokers’ exposure to secondhand smoke--United States, 1999-2012. MMWR Morb Mortal Wkly Rep.

[5] Yang S, Decker A, Kramer MS (2013). Exposure to parental smoking and child growth and development: a cohort study. BMC Pediatr.

[6] Martín-Pujol A, Fernández E, Schiaffino A (2013). Tobacco smoking, exposure to second-hand smoke, and asthma and wheezing in schoolchildren: a cross-sectional study. Acta Paediatr.

[7] Hymowitz N (2012). Cigarette Smoking and Lung Cancer: Pediatric Roots. Lung Cancer Int.

[8] Pistilli M, Howard VJ, Safford MM (2019). Association of secondhand tobacco smoke exposure during childhood on adult cardiovascular disease risk among never-smokers. Ann Epidemiol.

[9] Karadoğan D, Önal Ö, Kanbay Y (2018). Prevalence and determinants of smoking status among university students: Artvin Çoruh University sample. PLoS One.

[10] World Health Organization Global Adult Tobacco Survey Fact Sheet. In Chinese.

[11] Liu Y, Chen L (2011). New medical data and leadership on tobacco control in China. Lancet.

[12] Mbulo L, Palipudi KM, Andes L (2016). Secondhand smoke exposure at home among one billion children in 21 countries: findings from the Global Adult Tobacco Survey (GATS). Tob Control.

[13] Zheng ZL, Deng HY, Wu CP (2017). Secondhand smoke exposure of children at home and prevalence of parental smoking following implementation of the new tobacco control law in Macao. Public Health.

[14] Jeong SH, Jang BN, Kang SH, Joo JH, Park EC (2021). Association between parents’ smoking status and tobacco exposure in school-age children: assessment using major urine biomarkers. Sci Rep.

[15] Nanninga S, Lhachimi SK, Bolte G (2018). Impact of public smoking bans on children’s exposure to tobacco smoke at home: a systematic review and meta-analysis. BMC Public Health.

[16] Xie M, Jia C, Zhang Y (2020). Household Exposure to Secondhand Smoke among Chinese Children: Status, Determinants, and Co-Exposures. Int J Environ Res Public Health.

[17] Zhang L, Zhang Z, Cao Y (2022). Status and correlates of home smoking bans after the implementation of the smoke-free legislation in public places: A survey in Chongqing. Tob Induc Dis.

[18] Zheng P, Kegler MC, Berg CJ (2014). Correlates of smoke-free home policies in Shanghai, China. Biomed Res Int.

[19] Huang K, Chen H, Liao J (2016). Factors Associated with Complete Home Smoking Ban among Chinese Parents of Young Children. Int J Environ Res Public Health.

[20] Wei X, Zhang Z, Song X (2014). Household smoking restrictions related to secondhand smoke exposure in Guangdong, China: a population representative survey. Nicotine Tob Res.

[21] Fu M, Castellano Y, Tigova O (2019). Prevalence and correlates of different smoking bans in homes and cars among smokers in six countries of the EUREST-PLUS ITC Europe Surveys. Tob Induc Dis.

[22] World Health Organization 2018 China Adult Tobacco Survey Report.

[23] Park MB (2020). Living with parents who smoke predicts levels of toxicant exposure of children. Sci Rep.

[24] Agaku IT, Odani S, King BA, Armour BS (2019). Prevalence and correlates of secondhand smoke exposure in the home and in a vehicle among youth in the United States. Prev Med.

[25] Bayly JE, Bernat D, Porter L, O’Dare K, Choi K (2019). Prevalence and characteristics of secondhand smoke and secondhand vapour exposure among youth. Tob Control.

[26] National Health and Family Planning Commission of the People's Republic of China (2016). Guideline on China clinical smoking cessation (2015). Chinese Journal of Health Management.

[27] Zhou YH, Mak YW, Ho GWK (2019). Effectiveness of Interventions to Reduce Exposure to Parental Secondhand Smoke at Home among Children in China: A Systematic Review. Int J Environ Res Public Health.

[28] Lin H, Xiao D, Liu Z, Shi Q, Hajek P, Wang C (2019). National survey of smoking cessation provision in China. Tob Induc Dis.

[29] Zhang L, Li J, Lv Y (2020). Impact of tobacco control auxiliary resources on the 5As behavior in nursing interns: Self-reports from students. Tob Induc Dis.

[30] Hu M, Rich ZC, Luo D, Xiao S (2012). Cigarette sharing and gifting in rural China: a focus group study. Nicotine Tob Res.

[31] Huang LL, Thrasher JF, Jiang Y (2015). Impact of the ‘Giving Cigarettes is Giving Harm’ campaign on knowledge and attitudes of Chinese smokers. Tob Control.

